# Effects of tRNA-derived fragments and microRNAs regulatory network on pancreatic acinar intracellular trypsinogen activation

**DOI:** 10.1080/21655979.2021.2018880

**Published:** 2022-01-19

**Authors:** Hao Yang, Huairong Zhang, Zhuomiaoyu Chen, Yuan Wang, Bo Gao

**Affiliations:** a Department of General Surgery, The Second Affiliated Hospital of Harbin Medical University, Harbin, China; b Department of Endocrinology and Metabolism, Shanghai General Hospital, Shanghai Jiao Tong University School of Medicine, Shanghai, China; c Department of General Surgery, Peking University People’s Hospital, Beijing, China

**Keywords:** Acute pancreatitis, trypsinogen activation, small non-coding RNA, tRNA-derived fragments, tRF3-THr-AGT

## Abstract

Acute pancreatitis (AP) is a common gastrointestinal disease with substantial morbidity and mortality. Pancreatic acinar intracellular trypsinogen activation (PAITA) is an important event in the early stage of AP. The present study aimed to investigate the effects of tRNA-derived fragments (tRFs) and the microRNA regulatory network on pancreatic acinar intracellular trypsinogen activation (PAITA) and identify novel key targets in AP. Taurolithocholic acid 3-sulfate (TLC-S)-treated AR42J cells were used to establish a PAITA model. Twenty differentially expressed tRFs and 35 DE microRNAs were identified in PAITA through gene sequencing. Based on these genes, we established the tRF-mRNA and microRNA-mRNA regulatory networks by using bioinformatics methods. The networks revealed 29 hub microRNAs (e.g., Let-7 family, miR-21-3p.) and 19 hub tRFs (e.g., tRF3-Thr-AGT, i-tRF-Met-CAT) in PAITA. GO analysis showed that the functions of the two networks were similar and mainly enriched in RNA splicing, mRNA processing, and so on. tRF3-Thr-AGT, targeting Btg2, Cd44, Zbp1, etc., was significantly decreased in PAITA. Moreover, the trypsinogen activation level was increased significantly in the tRF3-Thr-AGT deficiency groups, but rescued by tRF3-Thr-AGT mimics. The results revealed that downregulated tRF3-Thr-AGT was involved in PAITA. This study provides potential novel targets for researching the underlying mechanisms of AP.

## Introduction

Acute pancreatitis is a common gastrointestinal disorder with increasing incidence [1]. The lack of specific treatment reminds us that the mechanism underlying acute pancreatitis is still awaiting more investigation. Self-digestion due to premature acinar intracellular activation of trypsinogen (PAITA) into trypsin is traditionally considered the trigger of acute pancreatitis. Abundant experimental models of acute pancreatitis consolidate this trypsin activation-centered theory of pancreatic injury. Trypsin within acinar cells can lead to cell apoptosis and induce inflammation in the pancreas [2]. Furthermore, trypsin-related mutations significantly alter the risk of acute pancreatitis in humans [3]. Therefore, exploring essential regulators during PAITA may aid in uncovering new therapeutic targets in acute pancreatitis.

MicroRNAs (miRNAs), the most famous small noncoding RNAs (sncRNAs), have emerged with multiple cellular functions in the past decade. A great number of reports show the complex regulatory mechanisms in inflammatory diseases by miRNAs through gene regulation, cell differentiation, apoptosis, and proliferation. It is not surprising that miRNAs exert various roles in AP. miR-135a and miR-22 were significantly upregulated in AP and promoted pancreatic acinar cell death by constraining their target kinases [4]. Another study found that miR-352 could activate intracellular trypsinogen and finally induce the occurrence of AP, which presented similar effects as sodium taurocholate [5]. Therefore, it is essential to discover the miRNA regulatory network in PAITA.

More recently, high-throughput sequencing revealed a new class of sncRNAs, tRNA-derived fragments, which are mapped to known transfer RNAs (tRNAs). tRNAs are classical ncRNAs responsible for conversion from genetic information to amino acid sequence information. Some tRNAs are involved in cell proliferation, metabolism, mitosis, and other biological processes [6]. The 5ʹ or 3ʹ halves of a mature tRNA (tiRNAs) generated by ribonuclease angiogenin (ANG) in humans in anticodon loops are capable of regulating protein translation [7]. Another class of sncRNAs derived from tRNA fragments (tRFs) with nucleotide sizes similar to those of miRNAs has been gradually uncovered in recent years. tRFs are universally found in the entire evolutionary tree with few well-documented functions. The expression of certain tRFs/tiRNAs may be dysregulated in cancer [8]. tRF-1001 was correlated with cell proliferation and the cell cycle in prostate cancer [9]. tRF-3 was involved in DNA damage response [10].

So far, whether tRF/tiRNA plays a role in inflammation, especially in acute pancreatitis, remains unknown. The aim of our study was to investigate the association between miRNA-mRNA and tRF/tiRNA-mRNA regulatory networks during PAITA. The secondary aim was to determine the key tRF/tiRNA involved in PAITA and its impact on trypsinogen activation.

## Materials and methods

### Cell culture and treatment

The rat pancreatic acinar cell line AR42J was obtained from the China Center for Type Culture Collection (Wuhan, China) and cultured in F12K medium (Sigma Aldrich; Merck KGaA) supplemented with 10% fetal bovine serum (Gibco; Thermo Fisher Scientific, Inc.) and 100 U/ml penicillin/streptomycin (Beyotime Institute of Biotechnology) in a 5% CO2 environment at 37°C. A total of 200 μM taurolithocholic acid 3-sulfate (TLC-S, Sigma Aldrich; Merck KGaA) was used to treat AR42J cells for 30 min at 37°C to establish the PAITA cell model [11].

### Detection of tRF/tiRNA, miRNA, and mRNA in PAITA

Total RNA was quantified using a NanoDrop ND-1000 instrument. Total RNA samples were first pretreated as follows to remove RNA modifications that interfered with small RNA-seq library construction: 3ʹ-aminoacyl (charged) deacylation to 3ʹ-OH for 3ʹ adaptor ligation, 3ʹ-cP (2ʹ,3ʹ-cyclic phosphate) removal to 3ʹ-OH for 3ʹ adaptor ligation, 5ʹ-OH (hydroxyl group) phosphorylation to 5ʹ-P for 5ʹ-adaptor ligation, and m1A and m3C demethylation for efficient reverse transcription. Then, pretreated total RNA was used to prepare the sequencing library in the following steps: 1) 3ʹ-adapter ligation; 2) 5ʹ-adapter ligation; 3) cDNA synthesis; 4) PCR amplification; and 5) size selection of 135 ~ 160 bp PCR amplified fragments (corresponding to 15 ~ 40 nt small RNA size range). The libraries were denatured as single-stranded DNA molecules, captured on Illumina flow cells, amplified in situ as sequencing clusters, and sequenced for 50 cycles on an Illumina NextSeq 500 system per the manufacturer’s instructions [12].

Image analysis and base calling were performed using Solexa pipeline v1.8 (Off-Line Base Caller software, v1.8). Sequencing quality was examined by FastQC software, and trimmed reads (pass Illumina quality filter, trimmed 3ʹ-adaptor bases by cutadapt) were aligned to mature-tRNA and pre-tRNA sequences from GtRNAdb using NovoAlign software (v2.07.11). The remaining reads were aligned to the transcriptome sequences (miRNAs). The expression profiling and differential expression of tRFs, tiRNAs, and known miRNAs were calculated based on normalized TPM [8]. Hierarchical clustering, scatter plots, and volcano plots were performed in R language for statistical computing and graphics for the differentially expressed (DE) tRFs, tiRNAs, and miRNAs. The sequencing data were uploaded to the GEO database (GSE181092).

In a published study [11], an Agilent 062716 Rat Microarray (8x60K; Agilent Technologies, Inc.) was used to detect the expression of mRNA, and then data analyses of the TLC-S group (trypsinogen activation model; n = 3) and control group (untreated AR42J cells; n = 3) were performed.

### miRNA and tRF/tiRNA regulatory network

The gene names were integrated by referring to the abbreviations in the Rat Genome Database (RGD). An mRNA with a fold change value ≥ 2.0 or ≤ 0.5 and P < 0.05 was considered differentially expressed. The differential multiples of miRNA transcription were compared. The DE tRF/tiRNAs and miRNAs were identified based on a differential multiple of 1.2 and P < 0.05. The rat 3ʹUTR sequence was downloaded from the ENSEMBL (http://asia.ensembl.org) database. Based on the miRanda (miRanda-3.3a) tool, we predicted the target genes of differentially expressed tRFs/tiRNAs and miRNAs. The selection threshold is the default score>140. Any case that met the criteria of either upregulation in the tRF/tiRNA and miRNA microarray and downregulation in the mRNA microarray for the predicted target gene or downregulation in the tRF/tiRNA and miRNA microarray and upregulation in the mRNA microarray for the predicted target gene was considered a potential miRNA and target gene mRNA pair. Based on the miRNA-mRNA pairs and tRF/tiRNA-mRNA, the regulatory network was constructed using Cytoscape software (Ver 2.6.3) [13].

### Function and pathway analysis

Gene Ontology (GO) functional and Kyoto Encyclopedia of Genes and Genomes (KEGG) pathway enrichment analyses for the genes regulated by each tRF/tiRNA and miRNA were performed using The Database for Annotation, Visualization and Integrated Discovery (DAVID) software (http://david.abcc.ncifcrf.gov/), and then the enriched functions and signaling pathways of the tRF/tiRNA and miRNA were annotated [14]. The specific function and pathway of each tRF/tiRNA and mRNA are shown with the R language ggplot package. The size of the bubble represents the number of enriched genes. The color represents the enrichment score (-lg(p value)); the redder the color is, the smaller the p value, and the greener the color is, the greater the p value.

### Cell transfection experiment

The Lipofectamine® RNAiMAX Transfection Reagent (Cat. No. 13778150, Thermo Fisher, USA) was prepared and added to a culture well containing 800 μL of culture medium with cells [12]. After 4–6 h of culture, the culture medium was replaced with fresh medium (containing fetal bovine serum and double antibodies), followed by continuous culturing for 48 h. The samples were divided into six experimental groups: control group, normally cultured AR42J cells; TLC-S group, nontransfected AR42J cells treated with 200 μM TLC-S for 30 min; tRF3-Thr-AGT NC+ TLC-S group, AR42J cells transfected with tRF3-Thr-AGT mimic-NC; tRF3-Thr-AGT mimics+ TLC-S group, AR42J cells transfected with tRF3-Thr-AGT mimics; tRF3-Thr-AGT NC group, AR42J cells transfected with tRF3-Thr-AGT inhibitor-NC; and tRF3-Thr-AGT inhibitor group, AR42J cells transfected with tRF3-Thr-AGT inhibitors. The sequence of the tRF3-Thr-AGT mimic was AUCCCAGCGGUGCCUCC, and the sequence of the tRF3-Thr-AGT inhibitor was GGAGGCACCGCUGGGAU (GenePharma, Shanghai, China). Moreover, angiogenin (ANG)-siRNA (Cat. No. AM16708, Thermo Fisher, USA) was used to inhibit ANG expression to verify that tRF3-Thr-AGT was a fragment derived from tRNA.

### Real-time PCR

RNA was extracted from the samples using total RNA TRIzol reagent (Cat No. DP405-02, Tiangen Biotech, Beijing, China). A PrimeScript™ RT reagent Kit with gDNA Eraser (Cat No. RR047B, TaKaRa, Japan) was used for cDNA reverse transcription with tRF3-Thr-AGT Real-time PCR primers (F: ATCCCAGCGGTGCCTCC; R: GGCCAACCGCGAGAAGATG) and U6 snRNA primers (F: CTGCGCAAGGATGACACGCAAATT; R: GGCCAACCGCGAGAAGATG) (GenePharma, Sh-anghai, China). An ABI 7500 fluorescence quantitative PCR analyzer (Applied Biosystems, USA) was used, and the relative quantification of the data was performed using the 2^−ΔΔ^Ct method [15].

### Laser confocal microscopy

The cells in the confocal dish were washed with phosphate buffered saline (PBS) 2–3 times; 150 μL of 4% paraformaldehyde was added for 15 min at room temperature to fix the cells. After being washed with PBS 2–3 times, the cells were incubated with PBS containing 2% Tween-20 for 10 min at room temperature. After being washed with PBS again 2–3 times, BZiPAR (CBZ-Ile-Pro-Arg) 2-rhodamine 110 (Molecular Probes, USA) staining solution was added at room temperature in the dark for 60 min. After being washed with PBS another 2–3 times, the cells were directly observed under a laser confocal microscope (A1R, Nikon, Japan) [11]. The ruler bar represents 9 μm.

### Flow cytometry

After the cells were digested and collected, 1 × 10^6^ cells from each group were centrifuged at 1,000 rpm for 5 min. The supernatant was discarded before 500 μL of 1× PBS was added to wash the sample two times, with centrifugation at 1,000 rpm for 5 min for each wash. The cells were then resuspended in 200 μL of BZiPARworking solution, followed by reaction at room temperature in the dark for 60 min. After centrifugation at 1,000 rpm for 5 min, the cells were resuspended in 500 μL of 1× PBS and evaluated using flow cytometry (Calibur II, BD Bioscience, USA). The cells were collected using CellQuest software, and the experimental data were analyzed using the Flowing software program. The state of the cells was observed with FSC/SSC, and the fluorescence intensity of the cells was observed with the FL1 fluorescence channel [11].

### Statistical analysis

All data are presented as the mean ± SD. Student’s t test or one-way ANOVA followed by Tukey’s post-hoc test was run when appropriate. Statistical analysis was performed using GraphPad Prism 5.0 software (GraphPad Software, Inc.). P < 0.05 was considered a statistically significant difference.

## Results

PAITA is the key trigger point in AP. In order to explore the role of non-coding small RNAs (tRF/tiRNA and miRNA) in PAITA. In this study, we used TLC-S to process AR42J cells to establish a PAITA model. Through gene sequencing, the differentially expressed miRNA, tRF and mRNA in PAITA were screened out. Then, miRNA-mRNA, tRF/tiRNA-miRNA and integrated regulatory network were established through bioinformatics methods. The networks revealed that tRF3-Thr-AGT has an important role in regulating PAITA. Further molecular biology experiments verified this result.

### DE mRNAs, tRF/tiRNAs and miRNAs in PAITA

**Figure 1.**
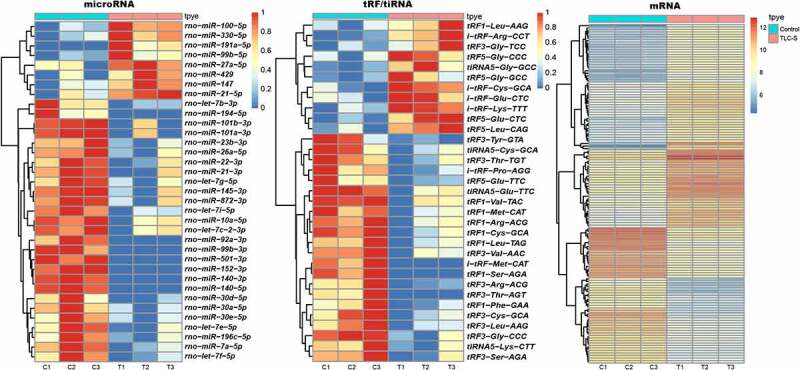
The heatmap of DE microRNA, tRF/tiRNA and mRNA.

Gene sequencing was used to identify the DE tRF/tiRNAs and miRNAs in PAITA. Compared with the control group, 20 DE tRF/tiRNAs and 35 DE miRNAs were observed in PAITA. Heatmaps of DE tRF/tiRNA and miRNA are presented in Figure 1. A total of 206 DE mRNAs were identified in PAITA by using a gene microarray. The heatmap of DE mRNA was presented in Figure 1. A summary of DE tRF/tiRNA, miRNA and mRNA expression is shown in Table 1.

**Table 1.** Number of DE mRNA, tRF/tiRNA and miRNA in PAITA

Expression	DE mRNA	DE miRNA	DE tRF/tiRNA
Upregulated	172	11	4
Downregulated	34	24	16
Sum	206	35	20

### The regulatory network of miRNA and tRF/tiRNA in PAITA

**Figure 2.**
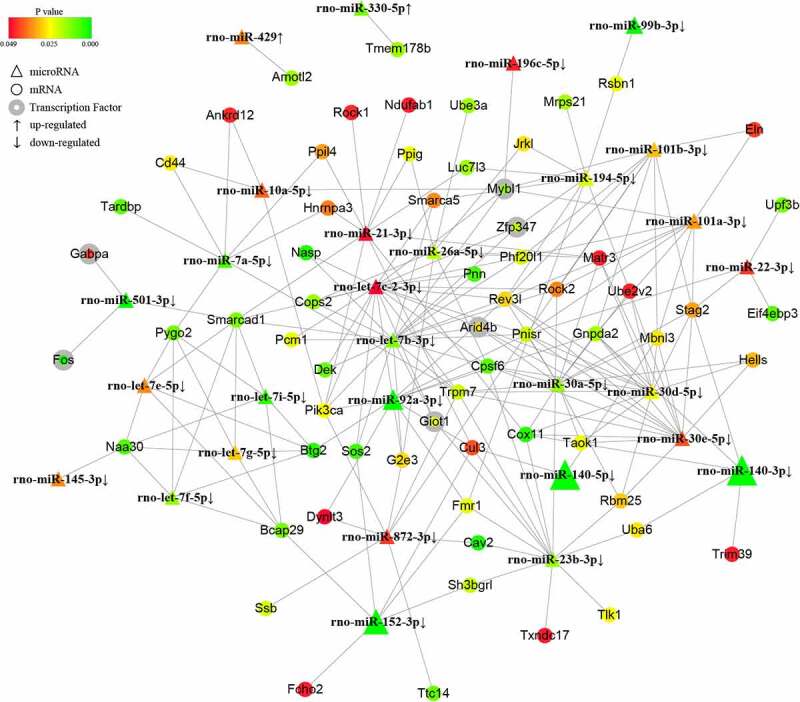
The microRNA-mRNA regulatory network in PAITA. Triangle represents microRNA. Circle represents mRNA. Circle with gray edge represents transcription factor. Color represents the p value of DE genes. Node size represents network degree of DE gene.

**Figure 3.**
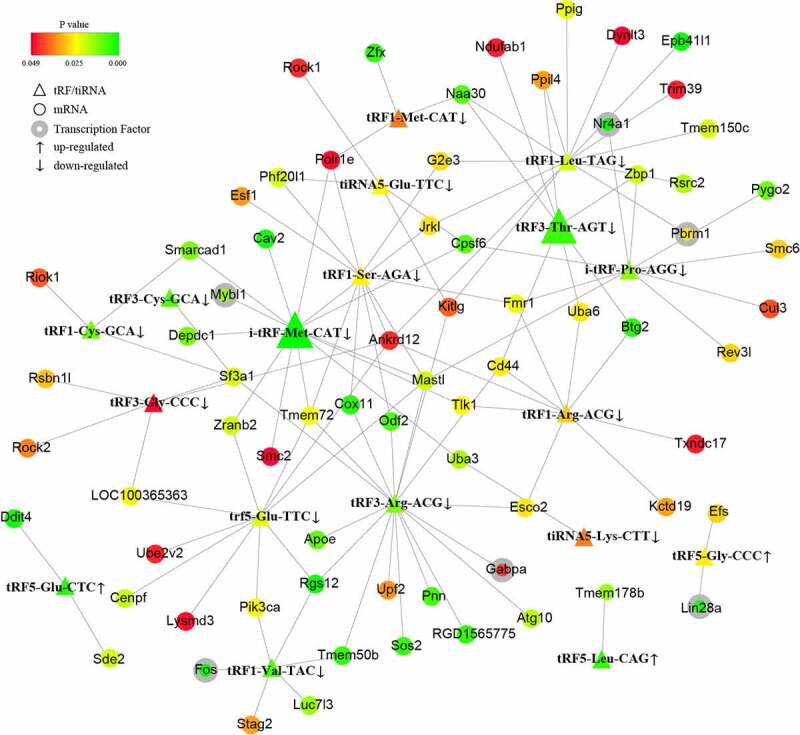
The tRF/tiRNA-mRNA regulatory network in PAITA. Triangle represents tRF/tiRNA. Circle represents mRNA. Circle with gray edge represents transcription factor. Color represents the p value of DE genes. Node size represents network degree of DE gene.

To further investigate the potential mechanisms of the noncoding small RNAs involved in PAITA, a bioinformatics database and Cytoscape software were used to establish the regulatory network of miRNA-mRNA and tRF/tiRNA-mRNA in PAITA. The color represents the p value; the greener the color is, the smaller the p value, and the redder the color is, the greater the p value. The node size represents the network degree of the DE gene. The results demonstrated 217 miRNA-mRNA pairs among 206 DE mRNAs and 35 DE miRNAs. The miRNA-mRNA regulatory network showed that rno-miR-140-3p, rno-miR-140-5p, rno-miR-92a-3p, and rno-miR-152-3p had high degrees and low P values (Figure 2). A total of 6 transcription factors were identified in the network (i.e., Mybl1, Fos, Giot1, Arid4b, Zfp347, and Gabpa). Moreover, a total of 111 tRF/tiRNA-mRNA pairs were identified among 206 DE mRNAs and 20 DE tRF/tiRNAs. The tRF/tiRNA-mRNA regulatory network revealed that i-tRF-Met-CAT and tRF3-Thr-AGT had the highest degree and the lowest P value (Figure 3). A total of 6 transcription factors were identified in the network (i.e., Mybl1, Fos, Nr4a1, Pbrm1, Lin28a, and Gabpa). Mybl1, Fos, and Gabpa were the overlapping transcription factors between the two networks. A combined network containing miRNAs, tRF/tiRNAs, mRNAs, and transcription factors was drawn to depict the underlying mechanism by sncRNAs in PAITA (Figure 6).

### GO and KEGG pathway analysis of the network in PAITA

**Figure 4.**
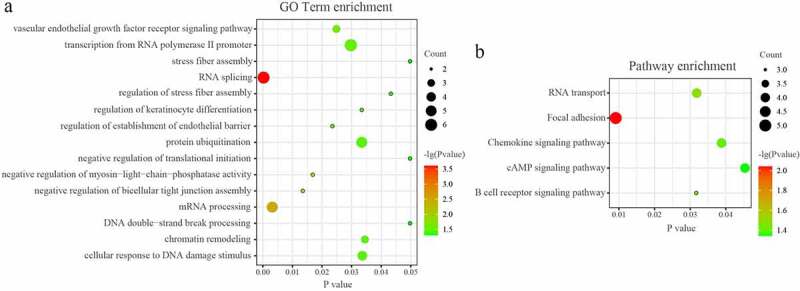
GO and KEGG pathway analysis of the microRNA-mRNA regulatory network in PAITA.

The DAVID database was used to analyze the enriched functions and pathways of genes in the network. GO analyses demonstrated that the genes in the miRNA-mRNA regulatory network were mainly enriched in ‘RNA splicing’, mRNA processing’, ‘protein ubiquitination’, ‘chromatin remodeling’, and ‘cellular response to DNA damage stimulus’. (*P* < 0.05; Figure 4(a)) KEGG analyses demonstrated that the genes in the miRNA-mRNA regulatory network were mainly enriched in ‘Focal adhesion’, ‘B cell receptor signaling pathway’, ‘RNA transport’, ‘Chemokine signaling pathway’, and ‘cAMP signaling pathway’. (*P* < 0.05; Figure 4(b))

**Figure 5.**
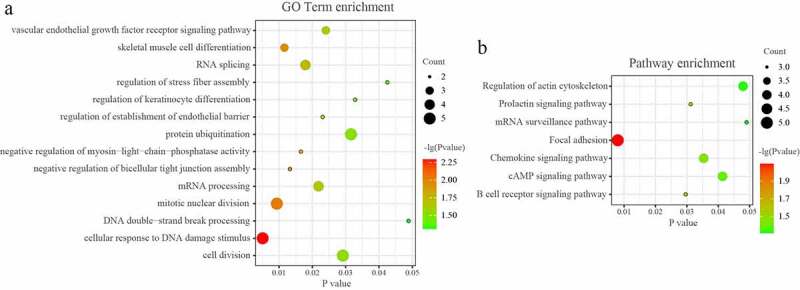
GO and KEGG pathway analysis of the tRF/tiRNA-mRNA regulatory network in PAITA.

GO analyses demonstrated that the genes in the tRF/tiRNA-mRNA regulatory network were mainly enriched in ‘mitotic nuclear division’, ‘cell division’, ‘RNA splicing’, and so on. (*P* < 0.05; Figure 5(a)) KEGG analyses demonstrated that the genes in the miRNA-mRNA regulatory network were mainly enriched in ‘Focal adhesion’, ‘Prolactin signaling pathway’, ‘Chemokine signaling pathway’, ‘Regulation of actin cytoskeleton’, ‘mRNA surveillance pathway’, and so on. (*P* < 0.05; Figure 5(b)).

**Figure 6.**
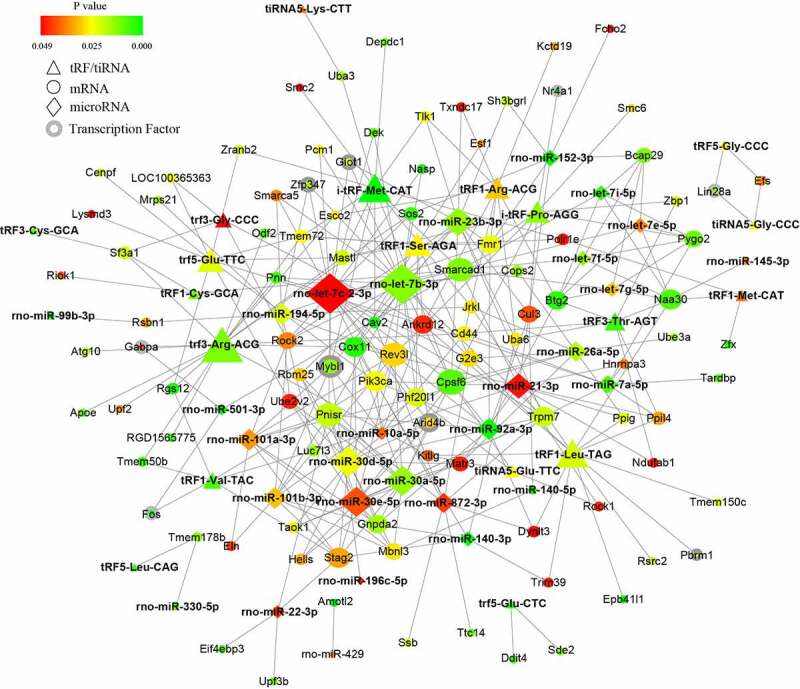
Combined microRNA and tRF/tiRNA regulatory network in PAITA.

### The different distribution of six tRF/tiRNA types in PAITA

**Figure 7.**
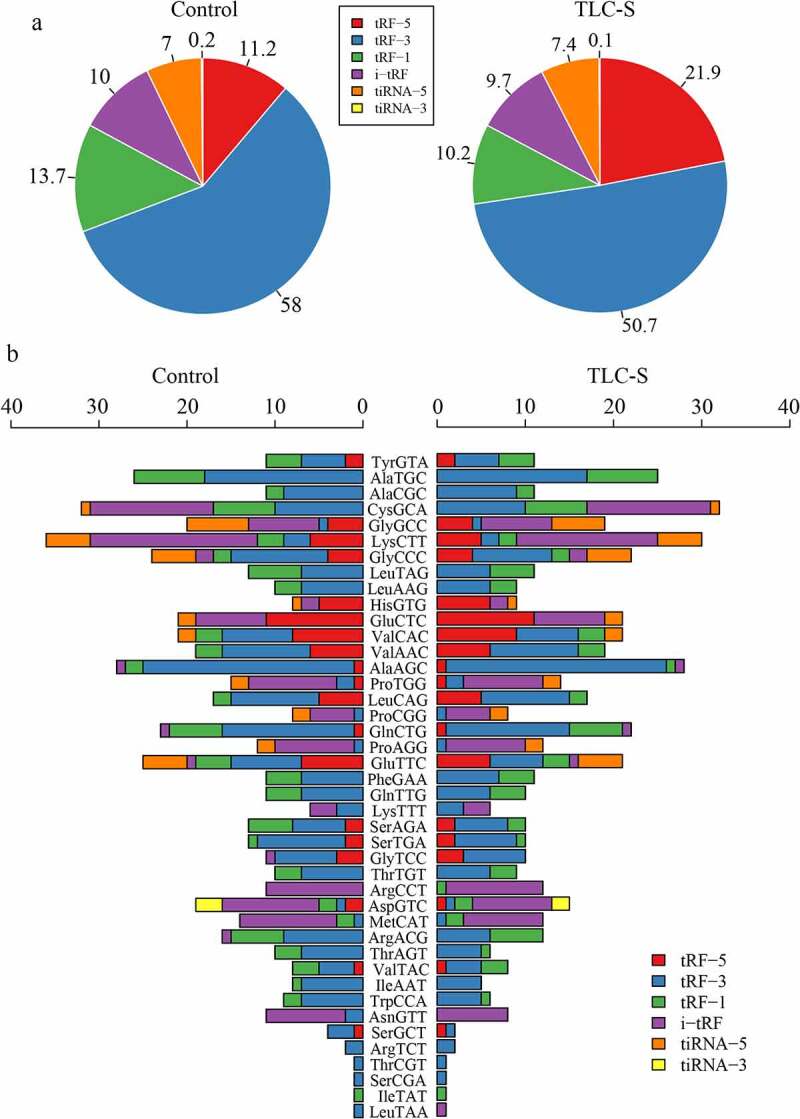
The distribution of six tRF/tiRNA types in PAITA. a, Overall distribution of six tRF/tiRNA types. b, Distribution of six tRF/tiRNA types in each tRNA.

tiRNAs are produced by specific cleavage in the anticodon loop under various stress conditions (29–50 nucleotides). The 5ʹ-halves are derived from the 5ʹ part of mature tRNA (tiRNA-5). The 3ʹ-halves are derived from the 3ʹ part of mature tRNA (tiRNA-3). tRFs are separated into 4 subtypes by their sites of origin in pre-tRNA or mature tRNA and are generally shorter than tiRNAs (16–28 nucleotides). tRF-5 is derived from the 5ʹ parts of mature tRNAs and is formed by cleavage in the D loop. tRF-3 corresponds to the 3ʹ parts of mature tRNAs containing 3ʹ CCA termini and is formed by cleavage at the T loop. tRF-1 originates from the beginning of the 3ʹ end flanking sequences with poly-U residues at the 3ʹ end. i-tRF, which does not belong to tRF-5, tRF-3, or tRF-1, is typically derived from the internal region of the mature tRNA sequence. Compared with the control group (11.2%), the proportion of tRF5 was significantly increased in PAITA (21.9%). However, the proportion of tRF3 was lower in the PAITA group (50.7%) than in the control group (58%). The network revealed that tRF3-Thr-AGT was the hub tRF in PAITA. The distribution proportion histogram showed that the proportion of tRF3 was significantly lower in the PAITA group than in the control group. (Figure 7(b))

### Verification of tRF3-Thr-AGT source

**Figure 8.**
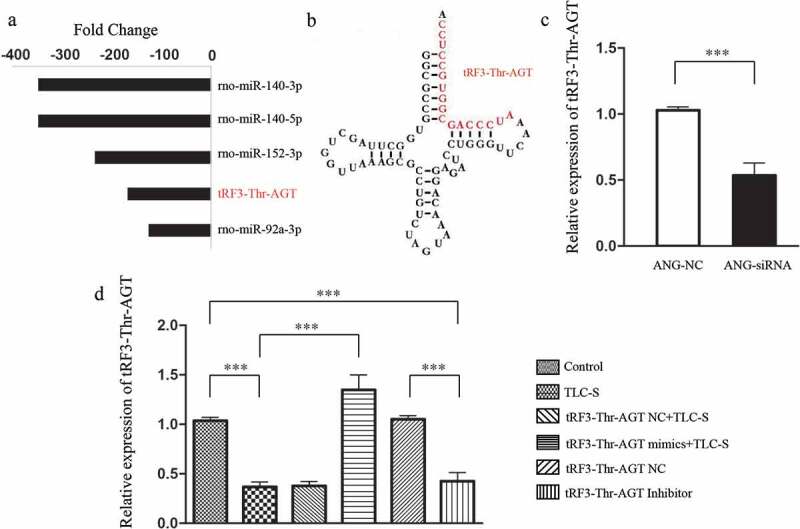
The expression of tRF3-Thr-AGT in PAITA and RNA intervention. a, The fold change of tRF3-Thr-AGT in PAITA. b, The sequence and location of tRF3-Thr-AGT in tRNA. c, Verification of tRF3-Thr-AGT source. d, The expression of tRF3-Thr-AGT in each group after RNA intervention. ***P < 0.001.

In the tRF/tiRNA-mRNA regulatory network, the expression level of tRF3-Thr-AGT in the PAITA group was 167 times lower than that in the control group (*P* < 0.01). Moreover, regulatory network analysis revealed that tRF3-Thr-AGT was a very important gene node with a high degree value, low p value, and the largest node size. The results suggested that tRF3-Thr-AGT may play an important role in PAITA. The base sequence of tRF3-Thr-AGT was AUCCCAGCGGUGCCUCC. Based on the sequence information, the nucleotide sequence may be derived from the tRNA fragment that transports the threonine (Thr) AGT codon (Figure 8(b)). After knocking down ANG with ANG-siRNA, the expression of tRF3-Thr-AGT in the ANG-siRNA group (1.028 ± 0.026) was significantly lower than that in the control group (0.529 ± 0.096). This result suggested that tRF3-Thr-AGT was derived from the tRNA spliced fragment.

### The expression of tRF3-Thr-AGT in each group after RNA intervention

As shown in Figure 8(d), the expression of tRF3-Thr-AGT in the TLC-S group was significantly lower than that in the control group (0.367 ± 0.052 vs. 1.035 ± 0.036, *P* < 0.001), which is consistent with the microarray results. The expression of tRF3-Thr-AGT in the tRF3-Thr-AGT mimics +TLC-S group was significantly higher than that in the TLC-S group (1.348 ± 0.152 vs. 0.367 ± 0.052, *P* < 0.001). The transfection of the tRF3-Thr-AGT negative control showed no significant impact on the expression of tRF3-Thr-AGT in the corresponding group. After normal AR42J cells were transfected with the tRF3-Thr-AGT inhibitor, the expression of tRF3-Thr-AGT in the tRF3-Thr-AGT inhibitor group was significantly lower than that in the tRF3-Thr-AGT NC group (0.425 ± 0.088 vs. 1.035 ± 0.036, *P* < 0.001).

### Trypsinogen activation in each group after RNA intervention

**Figure 9.**
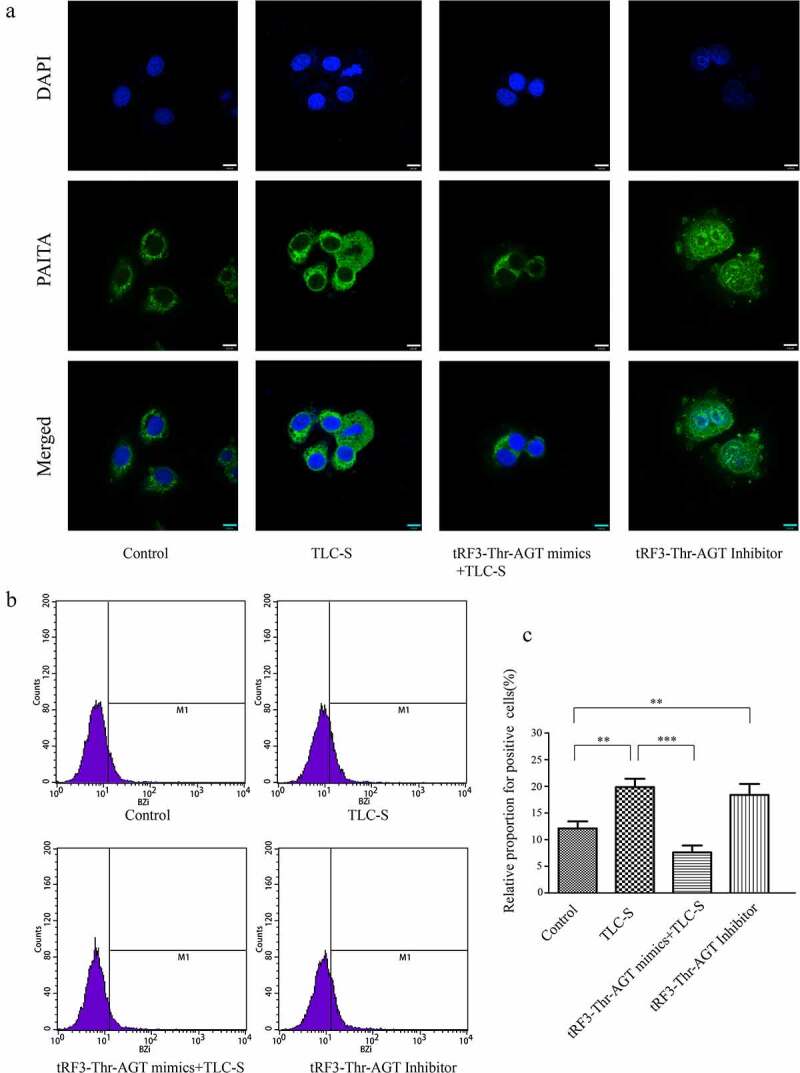
Trypsinogen activation in each group after RNA intervention. a, Image fromlaser confocal microscopy. b, Histogram of flow cytometry. c, Chart of statistics. **P < 0.01. ***P < 0.001.

BZiPAR is a specific substrate for serine protease. When BZiPAR is cleaved into two oligopeptides and reacts with activated trypsin, green fluorescence will be emitted. In this experiment, a quantitative study was performed using flow cytometry. The activation level of trypsinogen was evaluated by the percentage of green fluorescence-positive cells in total cells (Figure 9(b,c)). The results showed that the activation of trypsinogen in the TLC-S group was significantly higher than that of the control group (relative proportion for positive cells: 19.86% ± 1.58% vs. 12.10% ± 1.32%, P < 0.01). For the TLC-S group, trypsinogen activation in the tRF3-Thr-AGT mimics +TLC-S group was significantly lower than that in the TLC-S group (relative proportion of positive cells: 7.62% ± 1.29% vs. 19.86% ± 1.58%, P < 0.001). Although not treated with TLC-S, the tRF3-Thr-AGT inhibitor group showed significantly increased trypsinogen activation compared with the control group (relative proportion for positive cells: 18.38% ± 2.07% vs. 12.10% ± 1.32%, P < 0.01). The laser confocal microscopy image also showed this change trend (Figure 9(a)).

## Discussion

Accumulating evidence has shown the concrete view of PAITA during acute pancreatitis pathogenesis. The correlation between mutations in the gene encoding trypsinogen and hereditary acute pancreatitis is strong supportive evidence [16]. Many pharmacological agents can initiate PAITA by disrupting the synthesis, packaging, exocytosis of zymogen granules, and protein folding machinery of the endoplasmic reticulum. PAITA enhances acute pancreatitis in turn. However, the mechanism by which PAITA initiates acute pancreatitis is poorly documented.

We applied TLC-S to activate trypsinogen in the rat acinar cell line AR42J in vitro to mimic bile acid infusion acute pancreatitis. Twenty tRF/tiRNAs and 35 miRNAs were significantly altered by TLC-S-induced PAITA compared with untreated cells. Further bioinformatic analysis revealed two complex regulatory networks in tRF/tiRNA-mRNA and miRNA-mRNA. Functional analysis indicated that most genes involved in PAITA regulation were associated with cAMP, chemokines, mitotic nuclear division, and the actin cytoskeleton signaling pathway. Cylic AMP (cAMP) was first found to be induced intracellularly by trypsin almost 50 years ago [17]. cAMP agonists sensitized zymogen activation and increased cAMP abated acinar cell injury [18]. Perturbation of the actin cytoskeleton was also involved in amylase secretion by acinar cells [19] and was uniquely annotated in tRF pathway enrichment analysis. Cytoskeletal elements, including actin, are highly responsible for autophagosome formation, which is a common cell response in AP. These two networks have something in common regarding the GO term analysis. They were both significantly involved in the vascular endothelial growth factor receptor signaling pathway, RNA splicing, regulation of stress fiber assembly, keratinocyte differentiation, endothelial barrier establishment, protein ubiquitination, mRNA processing, and DNA damage response.

After merging the two networks with transcription factors, more detailed information was presented in terms of PAITA pathogenesis. The Let-7 family was frequently annotated in our predicted miRNA-tRF/tiRNA-mRNA-TF complex networks, including let-7e-5p, let-7i-5p, let-7 f-5p, let-7 g-5p, and let-7b-3p. Let-7 miRNA was found to be the first known human miRNA with high evolutionary conservation. The Let-7 family was able to negatively regulate RAS expression by binding to the 3ʹUTR [20]. RAS is predominantly involved in AP and pancreatic cancer. This partially explains why let-7 may be a vital player in AP initiation-PAITA. Let-7’s target mRNAs, including cav2, smarca5, Rock2, and target TF Arid4b, have been found to have various cellular functions in pancreatic cancer, but their roles in the field of AP are still unknown. miR-21-3p was found to not only modulate the inflammatory response to promote AP [21] but also positively correlate with the severity of AP in patients and aggravate pancreatitis in rats. miR-30a-5p, annotated in our networks, was previously reported to be essential for inflammatory response control in pancreatic acinar cells through the TGF-beta signaling pathway [22]. The abundant miRNAs and their target mRNAs or transcription factors predicted in our study warrant further investigation into AP pathogenesis.

tRFs and miRNAs have similar lengths and functional mechanisms. tRF3-Thr-AGT is an outstanding player in PAITA, indicated by the highest fold change and low P value. TLC-S inhibited its expression dramatically. We first found that it was derived from angiogenin-mediated tRNA cleavage by angiogenin-siRNA verification. To validate its involvement in PAITA, a tRF3-Thr-AGT mimic and inhibitor were applied. The tRF3-Thr-AGT mimic restored tRF3-Thr-AGT expression in the AR42J cell line after exposure to TLC-S. Accordingly, the tRF3-Thr-AGT mimic decreased TLC-S-induced trypsinogen activation. On the other hand, the tRF3-Thr-AGT inhibitor significantly decreased its expression compared with the control group. Green fluorescence was emitted again by inhibiting tRF3-Thr-AGT. Collectively, the expression level of tRF3-Thr-AGT was inversely correlated with trypsinogen activation. tRF3 is cleaved from the 3ʹ end of mature tRNAs by exonuclease, Dicer or ANG digestion in the TψC loop [23]. It has been linked with B cell lymphoma [24] and HIV viral infection [25]. However, its potential roles in inflammatory disease warrant deep investigation.

In our study, tRF3-Thr-AGT targeting the Btg2, Zbp1, and Cd44 genes was mapped to the regulatory network during PAITA. Btg2 was expressed at low levels in pancreatic cancer tissue compared with adjacent normal tissues [26]. Aberrantly expressed Btg2 was reported to regulate cell proliferation, the cell cycle, cell apoptosis, and tumorigenicity in several kinds of cancer [27,28]. Its impact on inflammation was first discussed in inflammatory bowel disease. High expression of Btg2 was mainly observed in the inflammatory rectal mucosa [29]. Fiedler et al. identified Btg2 mRNA as strongly overexpressed in the pancreas during the acute phase of pancreatitis. Moreover, the study suggested that Btg2 had antiapoptotic activity and might play a role in the control of apoptosis progression in AP. However, the role of Btg2 in PAITA is still not clear. Although recent studies have unveiled Zbp1 as a pathogen sensor and regulator of skin inflammation [30,31], its influence on PAITA is still unclear. Another downstream molecule of tRF3-Thr-AGT, Cd44, is relatively well documented. It has been implicated in the immune response and leukocyte recruitment, adhesion, and migration in inflammatory sites [32]. Abundant CD44 is present in inflammatory pancreatic cancers and may be related to progression [33]. All these data indirectly hinted that their upstream regulator, tRF3-Thr-AGT, might support its involvement in pancreatitis.

In conclusion, tRF3-Thr-AGT was inhibited in PAITA induced by TLC-S compared to normal pancreatic tissue. Exogenously enhanced tRF3-Thr-AGT expression eliminated trypsinogen activation. Reduction of tRF3-Thr-AGT by its inhibitor reactivated trypsinogen to an extent similar to TLC-S. Three targeted genes, Btg2, Zbp1, and Cd44, indirectly support the association between PAITA and tRF3-Thr-AGT in the mechanisms underlying acute pancreatitis initiation. Other significant tRF/tiRNAs, such as i-tRF-Met-CAT and miRNAs (let-7, miR-21-3p, etc.) were annotated to pave the way for future studies. Their specific functions in the inflammatory milieu are urgently warranted.

## Conclusion

Our study found that small noncoding RNA regulatory networks may play an important role in PAITA. The results of the network and molecular biology experiments revealed that downregulated tRF3-Thr-AGT was involved in PAITA.
